# Author Correction: Infectivity, susceptibility, and risk factors associated with SARS-CoV-2 transmission under intensive contact tracing in Hunan, China

**DOI:** 10.1038/s41467-021-23108-w

**Published:** 2021-04-30

**Authors:** Shixiong Hu, Wei Wang, Yan Wang, Maria Litvinova, Kaiwei Luo, Lingshuang Ren, Qianlai Sun, Xinghui Chen, Ge Zeng, Jing Li, Lu Liang, Zhihong Deng, Wen Zheng, Mei Li, Hao Yang, Jinxin Guo, Kai Wang, Xinhua Chen, Ziyan Liu, Han Yan, Huilin Shi, Zhiyuan Chen, Yonghong Zhou, Kaiyuan Sun, Alessandro Vespignani, Cécile Viboud, Lidong Gao, Marco Ajelli, Hongjie Yu

**Affiliations:** 1 Hunan Workstation for Emerging Infectious Disease Control and Prevention, Chinese Academy of Medical Sciences, Hunan Provincial Center for Disease Control and Prevention, Changsha, China; 2 School of Public Health, Fudan University, Key Laboratory of Public Health Safety, Ministry of Education, Shanghai, China; 3 Department of Epidemiology and Biostatistics, Indiana University School of Public Health, Bloomington, IN USA; 4 ISI Foundation, Turin, Italy; 5 West China School of Public Health and West China Fourth Hospital, Sichuan University, Chengdu, Sichuan China; 6 Division of International Epidemiology and Population Studies, Fogarty International Center, National Institutes of Health, Bethesda, MD USA; 7 Laboratory for the Modeling of Biological and Socio-technical Systems, Northeastern University, Boston, MA USA; 8 Department of Infectious Diseases, Huashan Hospital, Fudan University, Shanghai, China; 9 Shanghai Key Laboratory of Infectious Diseases and Biosafety Emergency Response, Shanghai, China; 10 Shanghai Institute of Infectious Disease and Biosecurity, Fudan University, Shanghai, China

**Keywords:** SARS-CoV-2, Viral epidemiology, Preventive medicine, Epidemiology

Correction to: *Nature Communications* 10.1038/s41467-021-21710-6, published online 9 March 2021.

The original version of this Article contained errors in Fig. 1 and Supplementary Fig. S3. The population density of Fig. 1b and Supplementary Figs. S3a, b, and c were given the incorrect units of “persons/km^2^”. This has been corrected to “persons/0.01 km^2^“. The colour scale of Fig. 1b and Supplementary Figs. S3a, b, and c were previously only labelled at the boundaries between groups, and only alternate labels were provided (the groups were labelled as “1”, “5”, “50” and “500”). This has been updated so that all groups have a separate label and the range is provided (the groups are now labelled as “[0.0, 1.0)”, “[1.0, 2.5)”, “[2.5, 5.0)”, “[5.0, 10.0)”, “[10.0, 25.0)”, “[25.0, 50.0)”, “[50.0, 100.0)”, “[100.0, 250.0)”, “[250.0, 500.0]”). The background colour of Fig. 1b was incorrectly shaded white, which suggested zero population density. The Figure has been updated with the correct shading of light yellow to indicate a population density of <1 person per 0.01 km^2^. The colour scale of Fig. 1c was previously only labelled at the boundaries between groups and only alternate labels were provided (the groups were labelled as “1”, “4”, “11” and “238”). This has been updated so that all groups have separate labels and ranges are provided (the groups are now labelled as “0”, “1-2”, “3”, “4-6”, “7-10”, “11-19”, “20-238”). These changes have been made in both the PDF and HTML versions of the Article.

## Supplementary information

Supplementary information.

